# Macular Morpho-Functional and Visual Pathways Functional Assessment in Patients with Spinocerebellar Type 1 Ataxia with or without Neurological Signs

**DOI:** 10.3390/jcm10225271

**Published:** 2021-11-12

**Authors:** Lucia Ziccardi, Ettore Cioffi, Lucilla Barbano, Valeria Gioiosa, Benedetto Falsini, Carlo Casali, Vincenzo Parisi

**Affiliations:** 1 IRCCS—Fondazione Bietti, Via Livenza 1, 00198 Rome, Italy; lucia.ziccardi@fondazionebietti.it (L.Z.); vincenzo.parisi@fondazionebietti.it (V.P.); 2 Department of Medical and Surgical Sciences and Biotechnologies, Sapienza University of Rome, Via Faggiana, 34, 04100 Latina, Italy; ettore.cioffi@uniroma1.it (E.C.); valeriagioiosa1991@gmail.com (V.G.); carlo.casali@uniroma.it (C.C.); 3 Ophthalmology Department, IRCCS—Fondazione Policlinico Universitario A. Gemelli—Catholic University, Largo Francesco Vito 1, 00168 Rome, Italy; benedetto.falsini@unicatt.it

**Keywords:** spinocerebellar ataxia type 1, multifocal electroretinogram, macular function, Sd-OCT, macular degeneration, visual pathways

## Abstract

Spinocerebellar ataxia type 1 (SCA-ATXN1) is an autosomal dominant, neurodegenerative disease, caused by CAG repeat expansion in the ataxin-1 gene (*ATXN1*). In isolated reports of patients with neurological signs [symptomatic patients (SP)], macular abnormalities have been described. However, no reports exist about macular anomalies in SCA1 subjects carrying the *ATXN1* mutation without neurological signs [not symptomatic carriers (NSC)]. Therefore, the main aim of our work was to evaluate whether the macular functional and morphological abnormalities could be detectable in SP, genetically confirmed and with neurological signs, as well as in SCA-ATXN1-NSC, harboring pathogenic CAG expansion in *ATXN1.* In addition, we investigated whether the macular involvement could be associated or not to an impairment of RGCs and of their fibers and of the neural conduction along the visual pathways. Herein, nine SCA-ATXN1 subjects (6 SP and 3 NSC) underwent the following examinations: visual acuity and chromatic test assessments, fundus oculi (FO) examination, macular and peripapillary retinal nerve fiber layer thickness (RNFL-T) analysis by Spectral domain-Optical Coherence Tomography (Sd-OCT) acquisition, multifocal electroretinogram (mfERG), pattern reversal electroretinogram (PERG) and visual evoked potentials (VEP) recordings. In four eyes of two SP, visual acuity reduction and chromatic abnormalities were observed; in three of them FO changes associated with macular thinning and outer retinal defects were also detected. In three NSC eyes, slight FO abnormalities were associated with qualitative macular morphological changes. By contrast, abnormal mfERG responses (exclusively from foveal and parafoveal areas) were detected in all SP and NSC (18 eyes). No abnormalities of PERG values, RNFL-T, and VEP responses were found, but in one SP, presenting abnormal papillo-macular bundle neural conduction. Results from our SCA-ATXN1 cohort suggest that a macular dysfunction, detectable by mfERG recordings, may occur in the overt disorder, and unexpectedly in the stage of the disease in which there is still an absence of neurological signs. In NSC, an exclusive dysfunction of preganglionic macular elements can be observed, and this is associated with both normal RGCs function and neural conduction along the visual pathways.

## 1. Introduction

Autosomal dominant spinocerebellar ataxias (ADSCAs) are neurodegenerative diseases which mainly affect the cerebellum and spinal cord but may involve various other systems [1]. The most common are the polyglutamine (polyQ) expansion spinocerebellar ataxias (SCAs), which result from a glutamine-encoding CAG repeat in the respective disease genes [2].

Spinocerebellar ataxia type 1 (SCA-ATXN1) is quite the most frequent among these rare diseases. It is caused by expanded CAG repeats (normal segments are 4–39 repeats, while the abnormal range is 40–80 repeats) in the in ataxin-1 gene (*ATXN1*), localized on chromosome 6p22.3 [3]. Its function is not completely understood yet, but it appears to be involved in regulating gene expression [4,5].

SCA-ATXN1 is characterized by an adult-onset (most frequently in the third or fourth decade) cerebellar syndrome. Non-cerebellar signs, such as pyramidal, cognitive, oculomotor, bulbar, and extrapyramidal are regularly found. In the overt SCA1 disease, as in other ADSCAs [6,7,8], relevant ocular impairment inducing a decrease in visual function has been reported. In details, macular dystrophy, outer retinal cavitation or foveal photoreceptoral disruption [9,10,11,12,13,14], as well as eye movement abnormalities or visual pathways disorders, as optic atrophy [13,15,16,17], have been described in patients with neurological signs (SP).

The macular area and the visual pathways can be morphologically and functionally evaluated by not invasive and reliable methodologies.

Macular structure and function can be assessed by Spectral domain Optical Coherence Tomography (Sd-OCT) and by multifocal electroretinogram (mfERG) recordings, respectively [18,19]. On the other hand, retinal ganglion cells (RGCs) axons, forming the optic nerve, can be morphologically studied by the analysis of the Sd-OCT retinal nerve fiber layer thickness (RNFL-T) [20], whereas the function of the innermost retinal layers (RGCs and their fibers) and of the neural conduction along the visual pathways can be assessed by Pattern ERG (PERG) [21,22] and Visual Evoked Potentials (VEP) [23,24] recordings, respectively.

Previous studies performed in visually impaired SCA-AXN1 patients [12,13], reported macular dysfunction assessed by mfERG recording [10,12], as well as macular morphological abnormalities detected by Sd-OCT examination. About the RGCs function, in absence of PERG results in SCA-ATXN1 patients, it seems that this matter was never investigated in this disease; regarding the RGC axon morphology, contrasting evidence has been reported, describing either reduced [13,17] or normal RNFL-T values [15].

Similarly, on the neural conduction along the visual pathways, normal [17,25] or impaired VEP findings were detected in mixed cohort of SCA1 and SCA2 patients [26,27,28].

Despite these evidence [6,10,12], there is a lack of information about the morpho-functional condition of the macular elements, the RGCs and of the optic pathways in those SCA1 patients, presenting the peculiar status of harboring the *ATXN1* gene mutation but with absence of any clinical neurological signs [not symptomatic carriers (NSC)] at this stage of disease.

Thus, in the present study, after neurological and genetic assessment, we performed an extensive ophthalmological evaluation of the macular function and structure, the RGCs function, the morphological condition of RGCs’ axons and the neural conduction along the visual pathways in SP and NSC.

Therefore, the main aim of our work was to evaluate whether the macular functional and morphological abnormalities could be detectable in SP and NSC. In addition, we investigated whether the macular involvement could be associated or not to an impairment of RGCs and of their fibers and of the neural conduction along the visual pathways.

## 2. Materials and Methods

### 2.1. Study Design and Participants

All research procedures described in this work have been performed in accordance with the ethical standards laid down in the Declaration of Helsinki of 1964 and its later amendments. The study protocol was approved by the local Institutional Review Board (Department of Medical and Surgical Sciences and Biotechnologies, Sapienza University of Rome) on 10 July 2019 and upon recruitment, informed consent after full explanation of the procedure was obtained from each subject enrolled in the study.

Nine subjects with genetically confirmed SCA-ATXN1 (4 male and 5 females, mean age 49.77 ± 8.56 years) were selected for the ophthalmological examination (see below) and enrolled at the Visual Neurophysiology and Neuro-ophthalmology Research Unit, IRCCS-Fondazione Bietti referred by the Department of Medical and Surgical Sciences and Biotechnologies, Sapienza University of Rome, Rome, Italy between September 2019 and June 2021.

In all SCA-ATXN1 subjects, extracted genomic blood DNA was analyzed by a customized targeted gene panel in next-generation sequencing to find CAG/CTG/CGG repeats in ATXN1 gene by TP-PCR [29] and Fluorescent Repeat-Primer PCR Assay [30].

In all SCA-ATXN1 subjects, neurological and ophthalmological examinations, comprehensive of an assessment of macular function-morphological condition, of the RGCs function, of the RGC axon function and morphology, of the neural conduction along the visual pathways were performed.

Based on the neurological examination (see below), six SCA-ATXN1 patients showed neurological signs (SP) and 3 SCA-ATXN1 subjects, harboring *ATXN1* gene mutation, showed absence of any clinical neurological signs at this stage of the disease (NSC).

A Group of selected 40 age-similar healthy subjects (mean age: 47.56 ± 6.63 years, 17 male and 23 females), providing 40 normal eyes, with best corrected visual acuity (BCVA) of 0.0 LogMAR (mean 0.0 ± 0.0) and absence of any macular morphological changes by fundoscopy and Sd-OCT evaluations, served as controls and provided the normal values for each of the below-described assessments.

### 2.2. Neurological Examination

All SCA-ATXN1 participants were evaluated using a standard protocol. Sex, age at onset, duration of the disease, and clinical manifestations (such as cerebellar ataxia, movement disorders, pyramidal signs, peripheral nerve signs, or cognitive dysfunction) were assessed.

For a semiquantitative analysis of ataxia associated signs the Scale for the Assessment and Rating of Ataxia (SARA) has also been used [31].

Occurrence and age of onset of visual complaints were investigated in all patients.

### 2.3. Ophthalmological Evaluation

All patients underwent a full ophthalmological evaluation including BCVA, intraocular pressure (IOP) measurement, color testing by monocular administration of Ishihara pseudoisochromatic plates (Kanehara Trading Inc., Tokyo, Japan), anterior segment observation by slit-lamp biomicroscopy and fundus examination by indirect ophthalmoscopy (by +90 Volk lens) after pupil dilation (1% Tropicamide drops).

Based on the following inclusion criteria

- absence of a mean refractive error > ±3.00 spherical equivalent;
- IOP less than 18 mmHg;
- absence of corneal or lens opacities;
- absence of square-wave jerks, saccadic intrusions and nystagmus in primary position of gaze that can influence the ability to maintain a stable fixation during the mfERG recordings (see below Section 2.5);
- Sd-OCT image with quality signal strength index > 40 (see below Section 2.6);
- absence of other systemic diseases (i.e., diabetes, systemic hypertension, rheumatologic disorders) or intake of drugs that may influence the retinal function.

### 2.4. Visual Acuity Assessment

BCVA was evaluated by the modified self-illuminated Early Treatment Diabetic Retinopathy Study (ETDRS) charts (Lighthouse, Low Vision Products, Long Island City, NY, USA) at the distance of 4 m. BCVA was measured as LogMAR.

### 2.5. Macular Functional Assessment

The function of the outer retina of the macular area was explored using VERIS mfERG system (VERIS Clinic TM version 4.9; Electro-Diagnostic Imaging, San Mateo, CA, USA) following the 2011 International Society for Clinical Electrophysiology of Vision (ISCEV) standards [32]. The multifocal stimulus, consisting of 61-scaled hexagons, was displayed on a high-resolution, black-and-white monitor (size, 32 cm width and 30 cm height) with a frame rate of 75 Hz. The array of hexagons subtended 20° of the visual field. Each hexagon was independently alternated between black (1 cd/m^2^) and white (200 cd/m^2^) according to a binary m-sequence. This resulted in a contrast of 99%. The luminance of the monitor screen and the central fixation cross (used as target) was 100 cd/m^2^. The m-sequence had 2^13−1^ elements, and total recording time was approximately 4 min. Total recording time was divided into eight segments. Between segments, the subject was allowed to rest for a few seconds. Focusing lenses were used when necessary. To maintain a stable fixation, a small red target, which was perceived by all subjects tested, was placed in the center of the stimulation field. A camera provided an image of the eye, which was displayed on the computer screen and the fixation was continuously monitored to exclude presence of small square-wave jerks, saccadic intrusions and nystagmus in primary position of gaze that may influence the fixation with relative not accurate mfERG recordings.

MfERGs were recorded in the presence of pupils that were maximally pharmacologically dilated with (1% Tropicamide drops) to a diameter of 7–8 mm. The cornea was anaesthetized with Benoxinate eye drops 0.4%. MfERGs were recorded between an active Dawson–Trick–Litzkow (DTL) contact electrode and a reference electrode (Ag/AgCl skin electrode placed on the correspondent outer canthi). A small Ag/AgCl skin ground electrode was placed at the center of the forehead. In the analysis of mfERG responses, we considered, for each obtained averaged response, the peak-to-peak response amplitude density (RAD) measured in nanoVolt/degree^2^ (nV/d^2^) between the first negative peak (N1) and the first positive peak (P1).

As already published in our previous works [33,34], we explored the bioelectrical responses derived from specific topographical retinal areas subdivided concentric annular retinal areas (rings) centered on the fovea. The averaged responses were obtained from five rings with increasing eccentricity from the fovea: from 0 to 2.5 degrees (ring 1, R1), from 2.5 to 5 degrees (ring 2, R2), from 5 to 10 degrees (ring 3, R3), from 10 to 15 degrees (ring 4, R4) and from 15 to 20 degrees (ring 5, R5).

**Figure 1:**
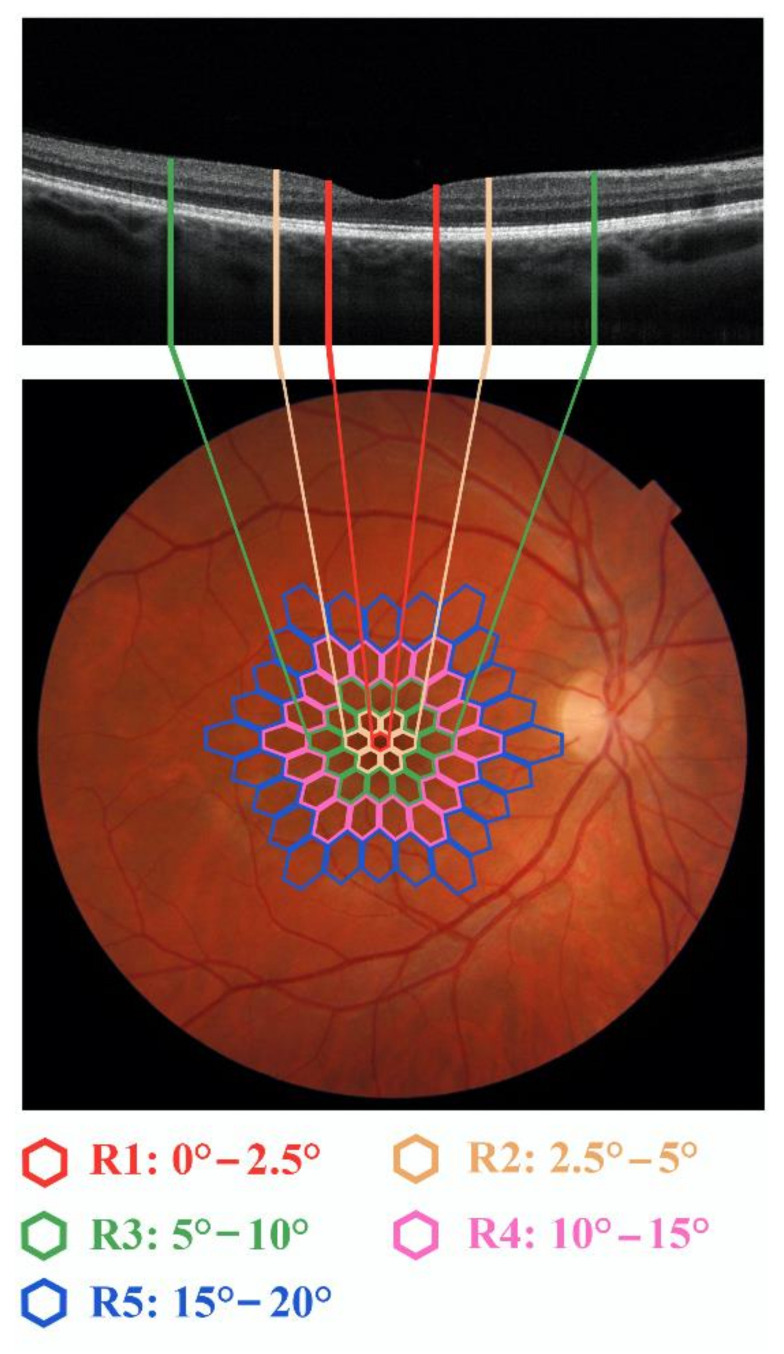
Fundus image from a representative Control (right eye) superimposed by the multifocal electroretinogram (mfERG) stimuli, provided by 61-scaled hexagons array subtending 20 degrees of the visual field. In the mfERG ring analysis we considered five rings, depicted in different colors, with increasing eccentricity from the fovea: from 0 to 2.5 degrees (ring 1, R1 in red), from 2.5 to 5 degrees (ring 2, R2 in brown), from 5 to 10 degrees (ring 3, R3 in green), from 10 to 15 degrees (ring 4, R4 in pink) and from 15 to 20 degrees (ring 5, R5 in blue). The central 3 mm (enclosed in the green bars) of a 6 mm macular line scan, obtained by Spectral domain-Optical Coherence Tomography (Sd-OCT), assesses the morphology of retinal structures enclosed in almost the 10 degrees of eccentricity from the fovea.

Figure 1 shows the topographical correspondence between mfERG examined areas (rings) and Sd-OCT macular scan superimposed (see below Section 2.6) on a fundus image from a Control subject.

### 2.6. Macular Morphological Assessment

The morphology of the macular area was explored in vivo by Sd-OCT (RTVue Model-RT100 version 6.3; Optovue Inc., Fremont, CA, USA), providing layer-by-layer objective measurements of anatomical structures. Sd-OCT scans were obtained in a dark room after pupil dilation with Tropicamide 1% drops and each scan was carefully reviewed for the accurate identification and segmentation of the retinal layers to exclude cases of failed segmentation. The Sd-OCT image quality signal strength index of the acquired scan was at least 40. APOSTEL recommendation according to the published criteria were followed [35]. Scans that did not fulfil the above criteria were excluded from the analysis. The RTVue-100 device uses a low-coherence light source centered at 840 nm with 50 nm bandwidth, which gives an axial resolution of 5 μm.

Using the MM5 protocol, we collected macular thickness and volume data from the ETDRS map. The MM5 grid scanning protocol consists of 11 horizontal lines with 5 mm scan length, 6 horizontal lines with 3 mm scan length, 11 vertical lines with 5 mm scan length, 6 vertical lines with 3 mm scan length each at 0.5 mm interval, all centered at the fovea [34]. The number of A-scans in long horizontal and vertical line are 668 and number of A-scans in short horizontal and vertical line are 400. This scan configuration provided an acquisition rate of 26,000 A-scans/second.

The segmentation algorithm of the MM5 scanning protocol also enables the automatic segmentation of macular thickness (MT, measured in microns) of the whole retina within 1 central mm (WR-MT), of inner retina (IR-MT) and of outer retina (OR-MT). In addition, it is possible to segment the macular volume (MV, measured in mm^3^) of the whole retina within 6 central mm (WR-MV), of inner retina (IR-MV) and of outer retina (OR-MV). The software automatically divides the inner and outer neurosensory retinas at the boundary between the inner nuclear layer (INL) and the outer plexiform layer (OPL). The OR encloses the OPL, the outer nuclear layer, and the photoreceptor layer. The IR examines the RNFL, the RGCs/inner plexiform layer (RGC/IPL), and the INL. The boundaries of the OR were the posterior of the OPL and the photoreceptor inner segment/outer segment junction. The following boundaries were identified for the IR segmentation: the inner limiting membrane and the posterior of the INL.

### 2.7. Assessment of RGCs Function and Neural Conduction along the Visual Pathways

Pattern Electroretinogram and Visual Evoked Potentials recordings were performed following our previous published methods [22,24,36].

Subjects were seated in a semi-dark, acoustically isolated room, in front of the display and surrounded by a uniform field of luminance of 5 candelas per meter squared, for monocular recordings. We used a visual stimulus of checkerboard pattern (contrast 80%, mean luminance 110 cd/m^2^) generated on a television monitor and reversed in contrast at the rate of 2 reversals per second. At the viewing distance of 114 cm, in the monitor screen subtending 23 degrees, the check edges subtended 60 min (60′) for VEP recordings and 15 min (15′) of visual angle. For VEP recordings, we used 2 different checkerboard patterns as suggested by the ISCEV standards [23] to obtain a prevalent activation of larger (60′ checks) or smaller (15′ checks) axons of the optic pathways [22,37]. PERGs were recorded in response to 15′ checks. A small fixation target, subtending a visual angle of approximately 0.5 degrees (estimated after considering spectacle-corrected individual refractive errors), was placed at the center of the pattern stimulus.

The setting for 15′ PERG recording was done by a small Ag/AgCl skin electrode placed over the lower eyelid. PERG signals were derived between the stimulated (active electrode) and the patched (reference electrode) eye using a previously described method. [38] The ground electrode was in Fpz. Interelectrode resistance was lower than 3000 ohms. The signal was amplified (gain 50,000), filtered (band-pass 1–30 Hz) and averaged with automatic rejection of artefacts (100 events free from artefacts were averaged for every trial) using Retimax Advanced Plus apparatus (CSO, Firenze, Italy). Analysis time was 250 milliseconds (ms). In the analysis of PERG responses, we considered the implicit time of P50 peak (P50 IT, measured in ms) and the peak-to-peak amplitude between the P50 and the N95 peaks, (PERG A, measured in microvolts).

The setting for VEP recordings was made of cup-shaped electrodes of Ag/AgCl were fixed with collodion in the following positions: active electrode in Oz, reference electrode in Fpz, and ground in the left arm. Interelectrode resistance was kept below 3000 ohms. The bioelectric signal was amplified (gain 20,000), filtered (band-pass 1–100 Hz) and averaged (200 events free from artefacts were averaged for every trial). Analysis time was 250 ms. We analyzed the IT of the peak P100, VEP P100 IT (measured in ms), and the peak-to-peak amplitude between the N75 and the P100 peaks, VEP N75-P100 amplitude (VEP A, measured in microvolts).

### 2.8. Morphological Evaluation of RGCs Axons

Peripapillary RNFL 3.45 protocol from RTVue Sd-OCT device (Model-RT100 version 6.3; Optovue Inc., Fremont, CA, USA) was used, following our previous work [39]. In the Sd-OCT analysis, we considered the average value of RNFL thickness (RNFL-T, measured in micros) of 4 quadrants.

### 2.9. Data Analysis

For BCVA, PERG, VEP and Sd-OCT parameters, 95% confidence limits (CL) were obtained from data detected in our Controls by calculating mean values + 2 SD for VEP and PERG ITs and mean values −2 SD for mfERG RADs, PERG A, VEP A, MT, MV, and RNFL-T. BCVA was considered to be abnormal for values greater than 0.0 LogMAR. The values detected in SP and NSC were considered to be “normal” when within the 95% CL and as “abnormal” when higher than 95% CL for VEP and PERG ITs and when lower than the 95% CL for mfERG RADs, PERG A, VEP A, MT, MV, and RNFL-T.

## 3. Results

The demographic, neurological and ophthalmological features of our SP (SP1, SP2, SP3, SP4, SP5, SP6) and NSC [NSC7 and NSC8 family members (children) of SP2, and NSC9 family member (brother) of SP4 and SP5] subjects are presented on Table 1.

### 3.1. BCVA Data

Among the six SP SCA-ATXN1 patients (SARA ranging from 8 to 29), visual acuity reduction and dyschromatopsia were detected in only two (SP1 and SP2, 4 of 12 eyes, 33.3%); in the remaining four SP patients (8 of 12 eyes, 66.6%), no visual symptoms (normal visual acuity and chromatic perception) were found.

In all three (NSC7, NSC8 and NSC9, 6 of 12 eyes, 100%) NSC subjects (SARA = 0), family members of the symptomatic ones, absence of visual symptoms was observed [BCVA was 0.00 LogMAR and chromatic perception was full (see Table 1)].

### 3.2. Macular Functional Data (mfERG Ring Analysis)

**Figure 2:**
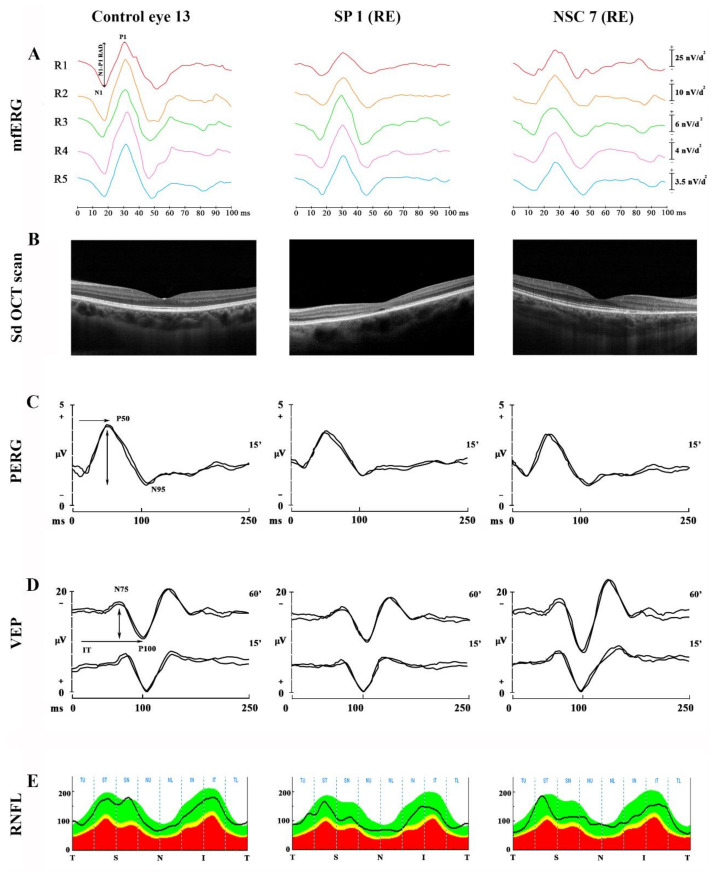
Examples of Multifocal electroretinogram (mfERG) recordings (**A**), Spectral Domain-Optical Coherence Tomography (Sd-OCT) scans (**B**), Pattern Electroretinogram (PERG) (**C**)**,** Visual Evoked Potentials (VEP) (**D**) and Retinal Nerve Fiber Layer (RNFL) thickness (**E**) performed in one representative control subject, in one SCA-ATXN1 symptomatic [SP1, right eye (RE)] patient and one not symptomatic [NSC7, right eye (RE] subject. In mfERG examples, the Ring analysis is reported, in which the N1-P1 (↨) response amplitude density (RAD, measured in nanoVolt/degree^2^—nV/deg^2^) was considered, obtained from five concentric annular retinal regions (rings) centered on the fovea: from 0 to 2.5 degrees (ring 1, R1), from 2.5 to 5 degrees (ring 2, R2), from 5 to 10 degrees (ring 3, R3), from 10 to 15 degrees (ring 4, R4) and from 15 to 20 degrees (ring 5, R5). In PERG and VEP examples, 60′ and 15′ refers to check edges subtending 60 min (60′) and 15 min (15′) of the visual angle for pattern reversal visual stimuli; P50 and N95 refer to the first positive and the second negative peak of PERG recordings, whose P50 implicit time (→) and P50-N95 peak-to-peak amplitude (↨) were considered; N75 and P100 refer to the first negative and the first positive peak of VEP recordings, whose P100 implicit time (→) and N75-P100 peak-to-peak amplitude (↨) were considered. In the RNFL analysis, T refers to temporal sector, S refers to superior sector, N refers to nasal sector and I refers to inferior sector. The average of the values detected in each sector was considered.

Examples of mfERG recordings performed in one representative control subject, in one SCA-ATXN1 symptomatic (SP1, right eye) patient and one not symptomatic (NSC7, right eye) subject are reported in Figure 2A.

Table 2 reports the individual values of N1-P1 RADs detected in the 5 rings (R1, R2, R3, R4 and R5) in SCA-ATXN1 SP and NSC subjects.

In all SP (12 of 12 eyes, 100%) abnormal functional responses recorded from the foveal area were found, as derived from the reduced mfERG R1 RADs. Additionally, two symptomatic patients (SP1 and SP2, 4 of 12 eyes, 33.3%), who also presented a reduction of BCVA, showed abnormal mfERG bioelectrical responses also in the perifoveal area, as suggested by the reduced R2 RAD.

Surprisingly, also all NSC subjects (6 of 6 eyes, 100%) showed abnormal functional responses from the foveal area (reduced mfERG R1 RADs) and two patients showed reduced RADs also in R2 (NSC7 only in right eye and NSC8 in both eyes, 3 of 6 eyes, 50%). Only in the right eye of NSC7(1 of 6 eyes, 16.6%) reduced RAD was recorded also in R3.

### 3.3. Macular Morphological (MT and MV) Data

Examples of Sd-OCT assessment performed in one representative control subject, in one SCA-ATXN1 symptomatic (SP1, right eye) patient and in one not symptomatic (NSC7, right eye) subject are reported in Figure 2B.

The individual MT and MV values detected in SCA-ATXN1 patients are reported in Table 3.

Among all SP, only two (SP1 and SP2 with the highest SARA score, 17 and 29, respectively) showed morphological impairment of the macular area. Sd-OCT analysis revealed reduction of WR-MV and of the relative segmented values (IR-MV and OR-MV) in 4 eyes (SP1 RE, SP1 LE, SP2 RE and SP2 LE), and reduced WR-MT and of the relative segmented values (IR-MT and OR-MT) in three of four eyes (SP1 RE, SP1 LE, and SP2 LE). In all NSC eyes, all values of Sd-OCT parameters were within normal limits.

By analyzing the Sd-OCT scans qualitatively, only SP1 and SP2 showed abnormalities, consisting of foveal cavitation between Retinal Pigmented Epithelium (RPE) and external limiting membrane (see Figure 2B for SP1 right eye), and/or RPE defects. The other SP patients showed normal macular profile at Sd-OCT scan.

Only in two NSC subjects qualitative abnormalities were found: monocular parafoveal RPE defect associated with ellipsoid zone (EZ) thinning in one subject (NSC7 right eye, see Figure 2B) and bilateral signs of slight RPE non-homogeneous reflectivity at Sd-OCT scan in another one (NSC9).

### 3.4. Data on RGCs Function (PERG) and Neural Conduction along the Visual Pathways (VEP)

Examples of PERG and VEP recordings performed in one representative control subject, in one SCA-ATXN1 SP (SP1, right eye) and in one NSC (NSC7, right eye) are reported in Figure 2C,D, respectively.

The individual values of PERG and VEP parameters detected in SCA-ATXN1 subjects are reported in Table 4.

None of SP or NSC subjects showed abnormal values of 15′ PERG parameters (P50 IT and P50-N95 Amplitude).

The values of 15′ and 60′ VEP parameters (P100 IT and N75-P100 Amplitude) were within the normal limits in all NSC and in 5 out of 6 SP (10 of 12 eyes, 88.3%); only in both eyes of SP2 (2 of 12 eyes, 16.6%) delayed 15′ VEP P100 ITs and reduced N75-P100 Amplitudes were detected.

### 3.5. RNFL-T Data

Examples of RNFL-T assessment performed in one representative control subjects, in one SP (SP1, right eye) patient and one NSC (NSC7, right eye) subject are reported in Figure 2E. The individual RNFL-T values detected in SCA-ATXN1 subjects are reported in Table 3.

The peripapillary Sd-OCT RNFL thickness analysis showed normal averaged values in all SP and NSC subjects.

## 4. Discussion

The main aim of our work was to assess in SCA-ATXN1 patients with neurological signs the presence or not of macular functional and morphological abnormalities and to explore whether similar macular involvement could be also detectable in SCA-ATXN1 patients without neurological signs.

In patients harboring pathologically expanded CAG sequences in *ATXN1* gene the penetrance, or appearance of neurological signs, is age-related, mainly dependent on the size of the expanded CAGs, and typically occurs in the third or fourth decade. Although unusually, patients can be found free of overt neurological signs at considerably later age. In our series, while one patient (NSC9) harbored a considerably shorter sequence than his symptomatic sister, explaining the divergence in terms of clinical manifestations, while two siblings (NSC7 and NSC8) harbored the same expanded CAG sequence as their affected mother. In such instances so far not understood genetic factors should be advocated to explain reduced age dependent penetrance [40].

In addition, we evaluated whether the macular involvement was associated or not to an impairment of RGCs and their fibers, and of neural conduction along the visual pathways.

### 4.1. Macular Functional and Morphological Changes in SP and NSC Patients

As main result of our study, all SP and NSC eyes (18 eyes from nine patients) showed a reduction of R1 and R2 mfERG RADs with normal R3, R4, and R5 RADs, suggesting a selective foveal (within the 0–2.5 degrees) and/or parafoveal (within the 2.5–5 degrees) dysfunction that was independent from the neurological phenotype.

About this topic, sporadic case reports reported controversial mfERG results. Previous studies [10,12] described in only three patients with severe neurological signs, features of visual acuity impairment and central retina dysfunction assessed by mfERG. Additionally, one single report by Hirose et al. [41] described localized abnormalities in the macula in a unique case of a symptomatic SCA-ATXN1 patient with reduced visual acuity. In a different cohort of SCA-ATXN1 symptomatic patients [12], one subject with normal visual acuity did not show any macular morpho-functional abnormality, whereas reduced mfERG responses were found in association with reduced visual acuity and foveal thinning in other subjects.

We evaluated the first-order kernel of the mfERG response, generated by preganglionic elements, located in outer and the middle retinal layers (photoreceptors and bipolar cells) [32]. Previous electrophysiological studies suggested that an abnormal mfERG provides strong evidence for preganglionic dysfunction [42,43,44]. Therefore, in SCA-ATXN1 patients, the reduced R1 and R2 RADs of the first-order kernel of mfERG responses may reflect a dysfunction of the more central macular preganglionic elements.

Unexpectedly, and this constitutes an interesting novel finding, non-symptomatic patients also showed an impairment of the preganglionic elements of the foveal and parafoveal macular area, as revealed by abnormal mfERG responses.

The mfERG has been already qualified as an important tool able to identify macular dysfunction in early stages of other ocular and non-ocular diseases, such as type 1 diabetes in absence of diabetic retinopathy [33], early age-related macular degeneration [45,46], SCA7 in absence of visual disorders [47] and early stage of multiple sclerosis without history of optic neuritis [48].

It is known that ataxin-1 is expressed not only in the brain tissue but also in non-neuronal tissues such as heart, skeletal muscle and liver [49]. It is likely that an extended polyQ chain of ataxin-1 could induce abnormal protein aggregation, or that aberrant interaction between the mutant ataxin-1 protein and other intracellular components could occur, resulting ultimately in substantial toxic effect and to cell loss [16,50]. The finding of foveal and parafoveal macular dysfunction with functional sparing of eccentric retinal areas in both manifest and in early ATXN1 pathology could support the hypothesis, although not yet confirmed, that ataxin-1 expression may occur in the cellular elements of the macula [50].

Indeed, while the macular function was impaired in SP and unexpectedly also in NSC, the morphological quantitative analysis of Sd-OCT between Groups (Controls vs. SCA-ATXN1 patients, enclosing both SP and NSC eyes) showed a significant MT reduction of WR and OR. Segmented IR-MT, IR-MV as well as WR-MV and OR-MV were not significantly different between Groups. According to our results, other studies observed reduced WR-MT [12,15] in symptomatic patients with visual impairment, and normal MT in a symptomatic patient without visual impairment [12]. Differently from our results, another report [13] described IR-MT reduction as marker of ganglion cells involvement. About the MV, only one report by Stricker et al. [17] reported a slight reduction of WR-MV. This result was, however, not statistically significant, as we also found when a statistical analysis of MV data was performed between our SCA-ATNX1 and Control Groups. No other evidence about retinal segmentation in SCA-ATXN1 patients was reported at the present.

When considering qualitative morphological findings, it is noticeable that photoreceptoral changes as well as EZ disruption were detected in three symptomatic eyes (SP1 RE, SP1 LE and SP2 RE) with more severe SARA score and visual acuity reduction. Similar evidence was already described by other authors only in symptomatic patients [10,12,13,41]. Lebranchu et al. [10] reported altered fovea lamination and abnormal spacing between RPE and external limiting membrane in four patients. Nishiguchi et al. [12] showed thinning of the central macula and disruption of the EZ. Hirose et al. [41] observed a complete absence of the interdigitation zone, locally absent EZ and RPE defects.

It is of interest that in our study, the EZ thinning and the parafoveal RPE defects were seen also in three not symptomatic eyes of two patients (NSC7 and NSC9). Our observations confirm the isolate evidence of a previous study by Lebranchu et al. [10], who described Sd-OCT abnormalities in one not symptomatic SCA-ATXN1 patient as “occult maculopathy” in association with SCA1-ATXN1 disease.

Considering the functional and morphological findings together in SP and NSC eyes, it is likely that the macular involvement may occur in the early stage of SCA-ATXN1 pathology.

This macular condition did not seem associated with the severity of the disease or to the VA impairment, being the outer retinal anomalies present also in NSC eyes.

### 4.2. Functional and Morphological Changes of RGCs and Their Axons

In all SP and NSC eyes of our study, we found normal values of 15′ PERG IT and A.

It is known that the PERG, recorded in response to high spatial frequencies visual stimuli (i.e., checks subtending 15′ of visual arc) mainly reflect the bioelectric activity of RGCs and their fibers, as suggested by studies performed in animal models [51] and in human pathologies [52]; however, a minor contribution of the retinal preganglionic elements cannot be entirely excluded from its genesis [53].

Therefore, our PERG data may suggest a normal function of the innermost retinal layers in our enrolled SCA-ATXN1 patients. However, since at the present there is lack of PERG studies in similar patients, our findings cannot be supported or contrasted by the available literature.

About the morphological RGCs involvement in SCA1 disease, an isolated report [13] described a significant reduction of RGCs and IPL thickness by Sd-OCT, not associated with information on functional data.

Overall, the significantly reduced WR-MT and OR-MT values detected in SCA-ATXN1 Group compared to Controls and the observed not statistically difference of IR-MT, WR-MV, IR-MV, OR-MV observed in concomitance with normal PERG values, but with abnormal mfERG responses, can be explained on the hypothesis that macular changes occur predominantly at the level of the outer retina.

The IR layers appear to be not morpho-functionally involved. Additionally, the large retinal area from which the PERG was recorded, might have obscured the foveal abnormalities found with the mfERG.

In addition, when comparing RNFL-T data between SCA-ATXN1 (enclosing SP and NSC patients) and Control Groups, no significant differences were found. This agrees with Pula et al. [15], who found normal RNFL-T values exclusively in SCA-ATXN1 patients, when analyzing data from a mixed cohort with SCA1, SCA2, and SCA3 genotype. Additionally, Nishiguchi et al. [12] confirmed the absence of RNFL-T changes in two observed patients. By contrast, Stricker et al. [17] showed RNFL-T reduction in the temporal sector, suggesting a prevalent impairment of the parvo-cellular axons from the papillo-macular bundle. Additionally, Oertel et al. [13] reported a reduction of RNFL-T as marker of possible optic atrophy. No evidence on RNFL-T was present in the literature about NSC SCA-ATXN1 patients.

It has been postulated that optic nerve atrophy could be due to a transcriptional dysregulation of ataxin-1, which finally causes RNFL neuroaxonal damage as part of a central nervous system degeneration [54]. Data from our cohort cannot confirm this hypothesis, and suggest, instead, functional and morphological integrity of the inner retinal elements.

### 4.3. Visual Pathways’ Function in SP and NSC Subjects

About the neural conduction along the large and the small axons of the optic pathways (evaluated by 60′ and 15′ VEP parameters, respectively) [39], normal values of IT and A were found in all SP and NSC eyes.

Only in one symptomatic patient (SP2, with the highest SARA score and with the worst macular condition but normal RNFL-T), we found bilateral increase of 15′ VEP IT (but normal 60′ VEP values), suggesting a selective delay of the neural conduction along the papillo-macular bundle axons.

Available data on VEP recordings are controversial, and biased by the not pure population of SCA, certainly because of the limited number of patients affected by this rare disease. Indeed, in symptomatic mixed cohorts of ADSCAs patients, including both SCA1 and SCA2 patients, VEP recordings were found abnormal in about 53% of the cases. However, no SCA1 patient showed increased P100 latency as well as reduction of N75-P100 amplitude [25]. Additionally, Stricker et al. [17] described normal VEP responses (without specification of the characteristics of the visual stimuli used) in nine SCA1 symptomatic patients, however, finding reduced RNFL-T on average as well as on temporal sector. They suggested a prevalent impairment of the parvo-cellular axons from the papillo-macular bundle. In a different study enclosing 19 SCA1 confirmed cases, VEP abnormalities were found in almost 42% of patients [26].

From our data, the normal VEP values recorded from all patients, but one, suggest a prevalent absence of delayed neural conduction along both large and small axons of the visual pathways in both SP and NSC eyes.

However, the increased 15′ VEP IT in only one SP patient suggests a selective impairment of the papillo-macular bundle, according to what already reported [17], for which can be hypothesized an involvement of antioxidant mechanism modulated by Ataxin 1 [55].

## 5. Conclusions

In conclusion, in this work we observed a macular dysfunction (reduction of foveal and parafoveal mfERG RADs) associated with reduced WR and OR macular thickness in SCA-ATNX1 patients. Normal RNFL-T, 60′ PEV and 15′ PERG responses were detected, and in only one SP patient abnormal 15′ VEP responses were observed.

We acknowledge that the small number of the study cohort, which was highly selected to avoid confounding factors impacting on macular function (see Materials and Methods) should represent a limitation of the study. However, to correctly perform mfERG recordings a normal target fixation is required, which may reduce the sample number.

Taken together, the observed remarkable data of macular dysfunction, detected in our cohort, without relevant neural conduction impairment along the visual pathways (except for the abnormal function of the papillo-macular bundle in the more affected symptomatic patient with evident macular changes) can suggest that a primary macular morpho-functional involvement in SCA1 phenotype may occur; this involvement may develop in the presence of neurological or ophthalmological signs and also in their absence.

The ophthalmological results obtained in our cohort seem not be related to the time elapsed from the onset of neurological signs (see Table 1 onset age neurological/visual signs), and this agrees with a previous report [56]. The evidence that macular impairment was detected in the youngest SP patient (SP3) and in all NSC subjects should suggest that the onset of macular dysfunction may be independent from age. It is possible that macular dysfunction is caused by a yet unrecognized pleiotropic effect of mutant ataxin-1 gene on photoreceptors [13,50].

## Figures and Tables

**Table 1:** Demographic, neurological and ophthalmological features of spinocerebellar ataxia type 1 patients with (SP) and without (NSC) neurological signs.

	SP1	SP2	SP3	SP4	SP5	SP6	NSC7	NSC8	NSC9
Age (Years)/Sex	50/M ^e^	68/F ^f^	36/M ^e^	51/F ^f^	50/F ^f^	50/F ^f^	43/M ^e^	47/F ^f^	53/M ^e^
**Family history**	Deceased mother affected by SCA1 ^g^	Mother of NSC7 and NSC8	No	Deceased mother with SCA1, sister of SP5 and NSC9	Deceased mother with SCA1, sister of SP4 and NSC9	Deceased father with SCA1	Mother (SP2) and sister (NSC8) affected by SCA1	Mother (SP2) and brother (NSC7) affected by SCA1	Deceased mother with SCA1, brother of SP4 and SP5
**SARA ^a^**	17	29	10	10	10	8	0	0	0
**CAG triplet expansion in ATXN1 gene** **(number)**	58	51	44	44	62	63	51	51	44
**Onset age** **Neurological/** **Visual Symptoms**	35/40	50/68	25/no visual symptoms	40/no visual symptoms	40/no visual symptoms	40/no visual symptoms	Not symptomatic/no visual symptoms	Not symptomatic/ no visual symptoms	Not symptomatic/no visual symptoms
**Neurological** **Signs**	impaired hand dexterity, mild dysarthria, dysphagia, gait ataxia	dysarthria, dysphagia, nystagmus, saccadic intrusions and severe gait ataxia	Mild dysarthria, gait ataxia, mild nystagmus	dysarthria, dysphagia, mild limb, gait ataxia	Mild dysarthria, dysphagia, hand dexterity, mild limb, gait ataxia	dysarthria, dysphagia, hand dexterity with alteration of writing, mild trunk, gait ataxia	None	None	None
**BCVA ^b^ RE ^c^/LE ^d^**	0.3/0.3	0.1/0.1	0.0/0.0	0.0/0.0	0.0/0.0	0.0/0.0	0.0/0.0	0.0/0.0	0.0/0.0
**Fundus Oculi** **examination RE ^c^** **LE ^d^**	Small parafoveal chorioretinal atrophy	Papillary pallor, macular dotted Dystrophy	Normal	Normal	Normal	Normal	Macular RPE dystrophy	Normal	Macular dystrophy
	Normal	Papillary pallor, macular dotted dystrophy	Normal	Normal	Normal	Normal	Normal	Normal	Macular dystrophy
**Ishihara charts** **RE ^c^** **LE ^d^**	15/22 16/22	2/22 6/22	22/22 22/22	22/22 22/22	22/22 22/22	22/22 22/22	22/22 22/22	22/22 22/22	22/22 22/22

^a^ SARA = Scale for the Assessment and Rating of Ataxia; ^b^ BCVA = Best Correct Visual Acuity measured in LogMAR; ^c^ RE = right eye; ^d^ LE = left eye; ^e^ M = male; ^f^ F = female; ^g^ SCA1 = spinocerebellar ataxia type 1.

**Table 2:** Multifocal electroretinogram data from spinocerebellar ataxia type 1 patients with (SP) and without (NSC) neurological signs.

	R1 ^a^ N1-P1 RAD ^f^ (nV/deg^2^) ^g^	R2 ^b^ N1-P1 RAD ^f^ (nV/deg^2^) ^g^	R3 ^c^ N1-P1 RAD ^f^ (nV/deg^2^) ^g^	R4 ^d^ N1-P1 RAD ^f^ (nV/deg^2^) ^g^	R5 ^e^ N1-P1 RAD ^f^ (nV/deg^2^) ^g^
SP1 RE ^h^	*35.85*	*17.31*	16.85	10.67	8.49
SP1 LE ^i^	*41.45*	*15.57*	15.47	12.52	8.77
SP2 RE ^h^	*33.78*	*14.06*	12.67	8.89	5.65
SP2 LE ^i^	*31.73*	*13.30*	14.77	12.11	9.81
SP3 RE ^h^	*45.28*	19.50	19.02	9.96	9.38
SP3 LE ^i^	*49.61*	20.87	15.29	11.34	8.76
SP4 RE ^h^	*36.68*	23.40	14.55	10.40	7.65
SP4 LE ^i^	*45.01*	28.09	11.70	10.16	7.80
SP5 RE ^h^	*47.40*	35.47	27.54	12.73	10.88
SP5 LE ^i^	*55.38*	28.79	20.60	13.82	8.82
SP6 RE ^h^	*37.29*	27.09	19.00	10.37	8.10
SP6 LE ^i^	*39.34*	23.31	17.78	11.64	8.08
NSC7 RE ^h^	*40.31*	*17.47*	*10.16*	7.57	6.26
NSC7 LE ^i^	*40.33*	24.78	13.09	8.99	6.21
NSC8 RE ^h^	*47.88*	*16.33*	10.74	7.47	6.01
NSC8 LE ^i^	*44.47*	*16.92*	12.29	6.50	4.70
NSC9 RE ^h^	*30.46*	23.28	14.61	10.77	6.69
NSC9 LE ^i^	*28.19*	26.55	16.60	8.13	4.73
95% CL ^l^	81.28	19.36	10.64	6.28	4.56

^a^ R1 = ring 1 (circular retinal area centered on the fovea: from 0 to 2.5 degrees); ^b^ R2 = ring 2 (concentric annular retinal area centered on the fovea: from 2.5 to 5 degrees); ^c^ R3 = ring 3 (concentric annular retinal area centered on the fovea: from 5 to 10 degrees); ^d^ R4 = ring 4 (concentric annular retinal area centered on the fovea: from 10 to 15 degrees); ^e^ R5 = ring 5 (concentric annular retinal area centered on the fovea: from 15 to 20 degrees); ^f^ RAD = response amplitude density; ^g^ nV/deg^2^ = nanoVolt/degrees^2^, ^h^ RE = right eyes; ^i^ LE = left eye; ^l^ CL 95%: Normal confidence limits obtained from control subjects by calculating mean values –2 standard deviations. On *italic* are reported the abnormal values.

**Table 3:** Spectral domain-Optical Coherence Tomography data in spinocerebellar ataxia type 1 subjects with (SP) and without (NSC) neurological signs.

	WR ^a^-MT ^b^ (μm) ^g^	IR ^c^-MT ^b^ (μm) ^g^	OR ^d^-MT ^b^ (μm) ^g^	WR ^a^-MV ^e^ (mm^3^)	IR ^c^-MV ^e^ (mm^3^)	OR ^d^-MV ^e^ (mm^3^)	RNFL-T ^f^ (μm) ^g^
SP1 RE ^h^	*168*	*51*	*117*	*4.782*	*1.886*	*2.884*	105.88
SP1 LE ^i^	*180*	*52*	*128*	*4.751*	*1.878*	*2.873*	110.82
SP2 RE ^h^	263	77	185	*4.642*	*1.655*	2.984	114.45
SP2 LE ^i^	*204*	*56*	*148*	*4.745*	*1.187*	*2.558*	105.97
SP3 RE ^h^	280	101	179	6.049	2.500	3.549	115.11
SP3 LE ^i^	259	86	173	6.14	2.548	3.592	120.86
SP4 RE ^h^	250	79	171	5.591	2.111	3.480	108.42
SP4 LE ^i^	249	76	174	5.672	2.169	3.502	122.73
SP5 RE ^h^	237	69	168	5.855	2.208	3.647	119.67
SP5 LE ^i^	251	79	171	5.929	2.357	3.572	117.79
SP6 RE ^h^	258	90	168	5.524	2.181	3.343	116.68
SP6 LE ^i^	246	70	176	5.549	2.201	3.348	118.33
NSC7 RE ^h^	260	81	180	6.299	2.618	3.681	111.03
NSC7 LE ^i^	253	81	172	5.759	2.252	3.508	108.41
NSC8 RE ^h^	265	88	177	6.204	2.571	3.632	120.83
NSC8 LE ^i^	271	81	190	6.177	2.546	3.361	125.32
NSC9 RE ^h^	242	70	164	5.718	2.239	3.479	116.08
NSC9 LE ^i^	254	86	168	5.810	2.308	3.502	116.91
95% CL ^l^	238.84	60.01	162.96	5.150	1.940	2.890	104.76

^a^ WR = whole retina; ^b^ MT = macular thickness; ^c^ IR = inner retina; ^d^ OR = outer retina; ^e^ MV = macular volume; ^f^ RNFL-T: retinal nerve fiber layer overall thickness; ^g^ μm = micron; ^h^ RE = right eyes; ^i^ LE = left eye; ^l^ CL 95%: Normal confidence limits obtained from control subjects by calculating mean values –2 standard deviations. On *italic* the abnormal values.

**Table 4:** Pattern Electroretinogram (PERG) and Visual Evoked Potentials (VEP) data from spinocerebellar ataxia type 1 patients with (SP) and without (NSC) neurological signs.

	15′ PERG ^a^		60′ VEP ^b^		15′ VEP ^a^	
	IT ^c^ (ms)	A ^d^ (µV) ^g^	IT ^e^ (ms)	A ^f^ (µV) ^g^	IT ^e^ (ms)	A ^f^ (µV) ^g^
SP1 RE ^h^	55	2.3	106	6.52	104	7.4
SP1 LE ^i^	56	2.4	108	5.91	104	7.5
SP2 RE ^h^	54	2.8	107	6.62	*121*	*5.1*
SP2 LE ^i^	53	3.0	103	5.9	*124*	*4.0*
SP3 RE ^h^	50	2.8	106	7.8	104	7.6
SP3 LE ^i^	53	2.3	105	6.6	109	7.8
SP4 RE ^h^	52	2.32	103	11.1	105	10.6
SP4 LE ^i^	56	2.6	104	7.9	104	9.7
SP5 RE ^h^	52	2.22	103	12.6	107	9.6
SP5 LE ^i^	55	3.2	106	16.2	106	7.8
SP6 RE ^h^	55	2.12	102	10.3	105	7.2
SP6 LE ^i^	57	2.19	104	13.22	104	6.2
NSC7 RE ^h^	56	2.7	100	11.5	99	8.6
NSC7 LE ^i^	53	2.4	100	8.5	97	6.9
NSC8 RE ^h^	57	2.21	103	6.5	104	9.7
NSC8 LE ^i^	54	2.24	106	13.3	102	10.3
NSC9 RE ^h^	52	2.34	107	7.7	108	8.6
NSC9 LE ^i^	55	2.62	107	6.32	107	7.7
95% CL ^l^	58.64	2.08	107.25	5.67	111.68	6.98

^a^ 15′ = responses obtained with checks edges subtending 15 min of arc; ^b^ 60′ = responses obtained with checks edges subtending 60 min of arc; ^c^ IT = P50 implicit time; ^d^ A: P50-N95 amplitude; ^e^ IT = P100 implicit time; ^f^ A: N75-P100 amplitude; ^g^ µV= microvolts; ^h^ RE = right eyes; ^i^ LE = left eye; ^l^ CL 95%: Normal confidence limits obtained from control subjects by calculating mean values +2 standard deviations for PERG IT and VEP IT and mean values –2 standard deviations for PERG A and VEP A. On *italic* are reported the abnormal values.

## Data Availability

Data are available upon request.

## Abbreviations

SCA-ATXN1	spinocerebellar ataxia type 1
ATXN1	ataxin-1 gene
FO	fundus oculi
Sd-OCT	spectral domain-optical coherence tomography
mfERG	multifocal electroretinogram
PERG	pattern reversal electroretinogram
VEP	visual evoked potentials
ADSCAs	autosomal dominant spinocerebellar ataxias
SCAs	spinocerebellar ataxias
SCA-ATXN7	spinocerebellar ataxia type 7
RGCs	retinal ganglion cells
BCVA	best corrected visual acuity
SARA	scale for the assessment and rating of ataxia
IOP	intraocular pressure
ETDRS	early treatment diabetic retinopathy study
DTL	Dawson–Trick–Litzkow
RAD	response amplitude density
MT	macular thickness
WR-MT	whole retina macular thickness
IR-MT	inner retina macular thickness
OR-MT	outer retina macular thickness
MV	macular volume
WR-MV	whole retina macular volume
IR-MV	inner retina macular volume
OR-MV	outer retina macular volume
INL	inner nuclear layer
OPL	outer plexiform layer
IPL	inner plexiform layer
IT	implicit time
A	Amplitude
RNFL-T	retinal nerve fibers layer thickness
SP	symptomatic patient with neurological signs
NSC	not symptomatic carriers without neurological signs
RE	right eye
LE	left eye
RPE	retinal pigmented epithelium
EZ	ellipsoid zone
